# Single amino acid substitution (G42E) in the receptor binding domain of mouse mammary tumour virus envelope protein facilitates infection of non-murine cells in a transferrin receptor 1-independent manner

**DOI:** 10.1186/s12977-015-0168-2

**Published:** 2015-05-16

**Authors:** Constantine James Konstantoulas, Benjamin Lamp, Tillman Hans Rumenapf, Stanislav Indik

**Affiliations:** Institute of Virology, University of Veterinary Medicine, Veterinaerplatz 1, 1210 Vienna, Austria

**Keywords:** Mouse mammary tumour virus, Transferrin receptor, Adaptation, Replication

## Abstract

**Background:**

Mouse mammary tumour virus (MMTV) is a betaretrovirus that infects rodent cells and uses mouse tranferrin receptor 1 (TfR1) for cell entry. Several MMTV strains have been shown to productively infect, in addition to murine cells, various heterologous cell lines including those of human origin, albeit less efficiently than murine cells. Furthermore, there have been reports that the continued passage of MMTV in heterologous cell lines gives rise to novel variants that are able to infect naive non-murine cells with higher efficiency than the parental virus.

**Results:**

We show that MMTV(C3H), like other MMTV strains, that had undergone a number of replication cycles in non-murine cells displayed an increased replication kinetic, as compared to parental virus, when applied on naive human cells. Sequence analysis of several replication kinetic variants and the parental virus, together with calculation of the ratio of non-synonymous to synonymous mutations at individual codons, revealed that several regions within the viral genome were under strong positive selection pressure during viral replication in human cells. The mutation responsible, at least in part, for the phenotypic change was subsequently mapped to the segment of *env* encoding the receptor binding site (F_40_HGFR_44_). Introduction of the identified mutation, leading to single amino acid substitution (G42E), into *egfp*-containing recombinant MMTV virions enhanced their ability to bind to and infect human cells. Interestingly, neither the replication kinetic mutant nor the parental virus required human TfR1 for infection. Knock-out of *TFR1* gene from the human genome did not decrease the susceptibility of Hs578T cells to virus infection. Furthermore, the expression of human TfR1, in contrast to mouse TfR1, did not enhance the susceptibility of MMTV-resistant Chinese hamster ovary cells. Thus, human TfR1 is dispensable for infection and another cell surface molecule mediates the MMTV entry into human cells.

**Conclusion:**

Taken together, our data explain the mechanism enabling MMTV to form ‘host-range variants’ in non-murine cells that has been known for a long time, the basis of which remained obscure. Our findings may expand our understanding of how viruses gain capability to cross species-specific barriers to infect new hosts.

**Electronic supplementary material:**

The online version of this article (doi:10.1186/s12977-015-0168-2) contains supplementary material, which is available to authorized users.

## Background

Cross-species transmission of viruses is well documented. Understanding how viruses infect and spread in new hosts is central to understanding the pathogenesis of infection and emergence of disease. Mouse mammary tumour virus (MMTV), the prototypical Type B betaretrovirus, infects *Muridae* rodents (of the *Mus* genus specifically) and is associated with mammary adenocarcinomas and T-cell lymphomas [1-4]. Mouse transferrin receptor 1 (mTfR1) is used by MMTV to initiate infection of murine cells [5]. The human ortholog (hTfR1), even though it has been reported to bind MMTV efficiently, does not serve as an entry receptor for MMTV [6]. Virus entry was blocked at a post-attachment phase due to a lack of internalization of MMTV-bound hTfR1 and subsequent trafficking to the late endosomes where fusion of membranes occurs [6]. Interestingly, although the virus cannot use hTfR1 for cell entry, several MMTV strains have been shown to productively infect, in addition to murine cells, various heterologous cell lines including those of human origin, albeit less efficiently than murine cells [7-11]. It has also been reported that MMTV sequences have been detected in human breast cancer and primary biliary cirrhosis specimens [12-17], as well as in canine and feline neoplastic and normal mammary tissue [11].

Recent reports also showed that MMTV-like viruses have once circulated more widely among rodents and other mammalian species. This belief comes from the identification of MMTV-like endogenous retroviruses (ERVs, fossils of now extinct viruses integrated into the genome of their host species) in rodent populations devoid of infectious MMTV and in other mammalian hosts of wide geographic and evolutionary diversity [18,19].

Additional evidence further supporting the notion that MMTV may be able to cross the species barrier and that MMTV–like viruses once circulated more widely among rodents is based on evolutionary analysis of rodent TfR1 amino acid residues that interact with MMTV-like virus envelope. These residues have undergone positive selection for mutations that compromise the interaction between the betaretrovirus entry glycoprotein and TfR1 [18]. At the same time, the entry glycoprotein receptor binding site (RBS; F_40_HGFR_44_ residues at the N-terminus-proximal region of the MMTV surface subunit (SU) domain [20]) has evolved to acquire compatibility with particular host TfR1 orthologs [18]. The molecular arms race between MMTV Env and rodent TfR1 driving endless rounds of ‘positive selection’ for mutations that affect interaction between the virus and host as well as above mentioned evidence support the concept that MMTV-like viruses once circulated more widely in nature and that they are particularly adept at overcoming cellular barrier preventing cross-species transmissions. Consistent with this model is the observation that continuous passage of MMTV in human or feline cell lines results in an adapted virus that infects various non-murine cells more efficiently that the parental virus [21,22]. At present, the nature of the adaptation mutation(s) responsible for this phenotypic change is unknown, however it can be envisaged that changes in the receptor binding site may allow the emergence of host range variants.

Such a scenario would be reminiscent of that reported for viruses belonging to two other virus families, *Parvoviridae* (canine and feline parvoviruses) and *Arenaviridae* (several rodent and human arenaviruses), which also use TfR1 as their primary cellular receptor to trigger their cellular entry. Canine parvovirus type 2a, is a host-range variant of the closely related feline parvovirus that gained access to dogs through the acquisition of only a few mutations in the capsid protein that allowed efficient binding of the virus to TfR1 expressed on the surface of canine cells [23,24]. It now appears that the transmission from cats to dogs was an indirect event involving passage through another carnivore species, racoon, which contributed to the adaptation of the capsid protein to canine TfR1 and the emergence of the canine parvovirus [25]. There is also indirect evidence suggesting that New World arenaviruses are able to, though minor changes in the arenavirus entry glycoprotein, expand their host range. Several of these viruses, which typically circulate in rodent species found in the Americas, are emerging into human populations, where they cause hemorrhagic fever and lethal disease [26,27]. Their zoonotic potential correlates with the ability to use the human TfR1 (hTfR1) in addition to their native rodent TfR1 [26]. Whereas clade B arenavirus that do not cause human disease (e.g. Amapari virus and Tacaribe virus) cannot utilize hTfR1 for cell entry, the pathogenic clade B arenaviruses effectively recognise the human ortholog, leading to the more efficient infection of human cells and, in turn, zoonotic transmission [28-30]. Interestingly, it has been shown that changes in as little as one amino acid residue of hTfR1 were sufficient for nonpathogenic arenaviruses to gain use of the receptor for cell entry [29]. Similarly, mutation of only a few amino acid residues enabled the conversion of the mouse TfR1 (mTfR1) to a compatible cell entry receptors for pathogenic arenaviruses [28]. From the aforementioned studies, it can be inferred that only minor changes in the receptor binding domain of GP1 may dramatically change arenavirus tropism.

Using both a wild-type and a recombinant *egfp*-containing virus carrying MMTV(C3H) Env, we have recently shown that MMTV(C3H), like other strains, is able to infect human cells, albeit less efficiently than mouse cells [31]. The established infection was, however, sufficient to enable virus spread to every cell in culture. Infectivity of the wild-type and MMTV(C3H)-Env-carrying MMTV recombinant virions was blocked by heat-inactivation and an inhibitor of reverse transcription, 3′-azido-3′-deoxythymidine (AZT), ruling out a non-specific mechanism of viral transfer [31]. Furthermore, infection requires an intact MMTV envelope protein, since neutralizing anti-MMTV antibodies blocked infectivity of both the wild-type and MMTV(C3H)-Env-carrying MMTV recombinant virions. Persistently infected Hs578T cells produced infectious virus particles capable of infecting naive human breast cells in culture, the infectivity of which could be blocked by neutralizing anti-MMTV antibodies, heat-inactivation and AZT, demonstrating that virus particles released by the persistently infected Hs578T cells are antigenically related to the virus produced from murine cells and showing that the presence of proviral DNA in infected cells is due to an authentic, receptor-mediated and reverse transcriptase-dependent infection process [31].

Herein, we analyse whether the replication of MMTV(C3H) in human cells results in sequence alterations that facilitate its spread and survival in the new host. Consistent with previous reports we show that the replication of MMTV in non-murine cells leads to the formation of variants that replicate faster in human cells. Furthermore, we demonstrate that the mutation responsible, at least in part, for the phenotypic change is located in the receptor binding site of the viral glycoprotein. Finally, we present evidence showing that the human TfR1 is not required for more efficient infection of human cells with the replication kinetic mutant, suggesting that the virus has adapted to use another cell surface molecule for cell entry.

## Results

### MMTV(C3H) particles released by the persistently infected human cells exhibit an accelerated cell-to-cell spread phenotype in human cells

Previously, we have shown that wild-type MMTV(C3H) and a recombinant EGFP containing virus carrying MMTV(C3H) Env could infect cultured human cells via a specific interaction of the viral envelope with the cell surface receptor [31]. Additionally, the wild-type virus [MMTV(C3H)], harvested from cell culture fluids of an MMTV-induced mouse mammary tumour cell line (Mm5MT), was able to replicate in a human mammary epithelial cell line (Hs578T), ultimately leading to the infection of every cell in culture [31]. These persistently infected human Hs578T cells produced infectious virus particles [designated MMTV(C3H)hp1] capable of infecting naive human breast cells in culture. The infectivity of MMTV(C3H)hp1 was blocked by neutralizing anti-MMTV antibodies, demonstrating that virus particles released by the persistently infected human cells are antigenically related to the virus produced from murine cells [31]. Here we have extended this earlier work by addressing the question of whether MMTV(C3H)hp1 is able to productively replicate in human breast cells and whether the replication kinetics of the virus in non-murine cells changed relative to the parental virus.

For this set of experiments, cell free virus obtained from cell culture fluids of human cells persistently infected with MMTV(C3H) (6 weeks post infection) or wild-type virus obtained from cell culture fluids of an MMTV induced mouse mammary tumour cell line derived from the C3H/HeN mouse strain (Mm5MT) [32] was used to infect naive, uninfected Hs578T cells. Prior to infection, virus particle titers were determined by real-time TaqMan RT-PCR targeting the 5′ end of *env* and normalized virus levels were used for infection of human cells. Quantification of proviral DNA one week post infection, using real-time TaqMan PCR, showed approximately equivalent proviral load in the infected cells. Next, the cells were cultured in the presence or absence of DEX (10^−6^ M) and gDNA was extracted at regular time points.

**Figure 1:**
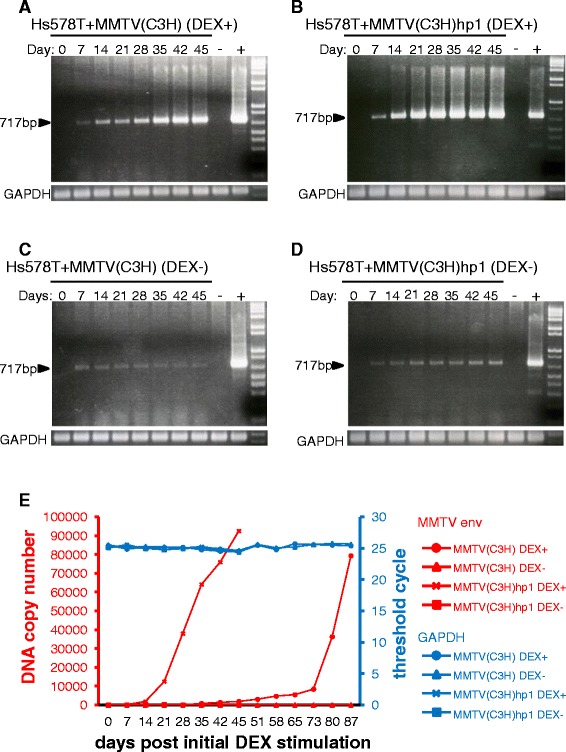
Quantification of proviral DNA in cell lysates of first and second round infected human Hs578T cells during a time-course experiment. Hs578T cells infected with either MMTV(C3H) **(A & C)** or MMTV(C3H)hp1 **(B & D)** were cultured in the presence or absence of 10^−6^ M DEX. Genomic DNA extracted from infected cells at the indicated time points and semi-quantitative PCR performed. -ve: non-transduced Hs578T cells. +ve: producer cells. Equal DNA loading was controlled in the PCR assay with GAPDH-specific primers (bottom panels). Real-time TaqMan PCR was performed for an accurate quantification of proviral loads in infected Hs578T cells **(E)**, during the time-course experiment. Equal loading of the PCR reactions were quantified in a real-time TaqMan PCR specific for the APOB gene.

In agreement with our previously published data [31], increasing levels of proviral DNA were detected within Hs578T cells infected with MMTV(C3H) over time (Figure 1A & E), as assessed by semi-quantitative (targeting the MMTV LTR-*gag* region) and quantitative (targeting the 5′ end of the *env* coding region) PCR. Importantly, an increase in proviral DNA levels was also detected for cells infected with MMTV(C3H)hp1 (Figure 1B & E). Interestingly, the infectious particles released by the persistently infected human cells, MMTV(C3H)hp1, showed an increased replication kinetic in human cells compared to viruses produced from Mm5MT cells. Specifically, all Hs578T cells infected with MMTV(C3H)hp1 carry a copy of the proviral DNA at 21 days post infection (Figure 1E). In contrast, Hs578T cells infected with MMTV(C3H) need to be cultured for at least 80 days before all cells carry a copy of the proviral DNA (Figure 1E). The increase was dependent on the presence of DEX in the cultivation medium, since no noticeable augmentation of proviral DNA levels was detected in cells cultured in the absence of DEX (Figure 1C, D and E).

Collectively, these findings show that MMTV(C3H)hp1 virus produced from persistently infected human cells is not only able to replicate in naive human cells, but also exhibits a faster replication kinetic in human cells.

### Sequence analysis of the virus replicating in human cells

Next, we determined twelve complete MMTV(C3H)hp1 proviral sequences (minus the *gag* gene, which contains a sequence precluding cloning and growth in *E. coli*) and compared them to MMTV(C3H) sequences identified in virus preparations from murine mammary carcinoma cells. No recombination events with sequences of human origin were identified in the proviral sequences derived from viruses that have undergone several replication cycles in human cells. Similarly, there was no evidence to suggest that the virus capable of replicating in human cells was a variant resulting from recombination with mouse genomic sequences, including the endogenous Mtvs.

**Figure 2:**
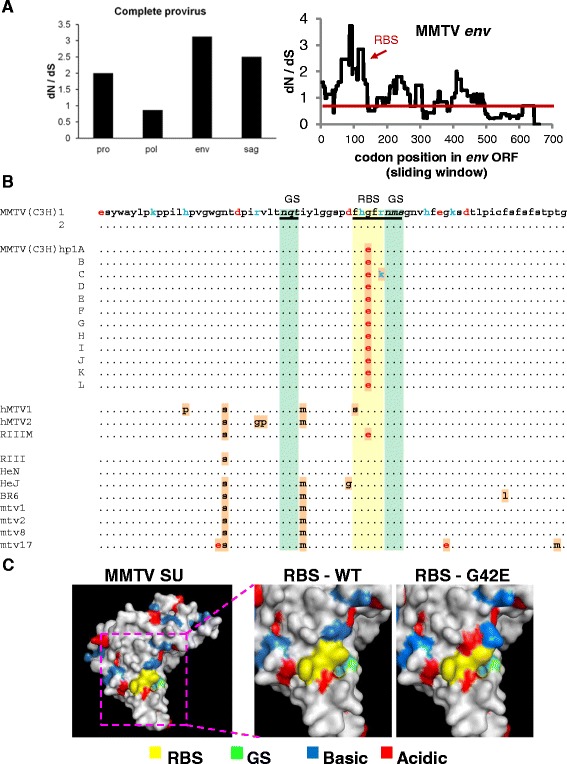
Sequence analysis of MMTV(C3H)hp1 from the second round infected Hs578T cells. Genomic DNA isolated from Hs578T cells 2 weeks after infection with MMTV(C3H)hp1 or cDNA synthesised from RNA extracted from filtered producer cell (Mm5MT) culture medium was used for amplification and cloning of the entire MMTV genome, minus the *gag* gene. **(A)** The ratio between the number of non-synonymous mutations per non-synonymous site (dN) and synonymous mutations per synonymous site (dS) was calculated using the Tamura-Nei method implemented in the MEGA software package that estimates the number of non-synonymous and synonymous changes that have occurred at each codon throughout evolution [33]. In the right panel, the dN/dS ratio for subregions in the MMTV *env* analysed using a sliding window of 100 codons is shown. **(B)** Sequence alignment of amino acid residues 1 through 72 of MMTV SU of two virus clones [MMTV(C3H)] and twelve proviral clones [MMTV(C3H)hp1] were aligned. *Mtv-1* (AF228550.1), *Mtv-2*, *Mtv-8* (M22028.1), *Mtv-17* (AF263910.1), MMTV(C3H-HeN) (AF228552.1), MMTV(C3H-HeJ) (AF2258551.1), MMTV(RIII) (AF071010.1) and MMTV(BR6) (M15122.1) sequences were also included in the alignment. The coordinates of the MMTV Env amino acid sequence are according to Zhang et al. [20]. Potential N-glycosylation sites (GS) are highlighted in light green and RBS in yellow. **(C)** Predicted 3D structure of the MMTV SU in space-filled form generated using Swiss-Model is shown. The RBS is indicated in yellow, the glycosylation site in green, basic residues in blue and acidic residues in red. In the right panel, the RBS is enlarged and the charge change caused by G to E substitution at position 42 is highlighted.

Several nucleotide substitutions were detected in the *pro* (a portion), *pol*, *env* and *sag* genes *(data not shown)*. The greatest number of point mutations was found in the Env coding region. Next, we sought to analyze whether selection pressures shaped the genetic variation during cultivation in human cells. Calculation of the ratio between the number of non-synonymous mutations per non-synonymous site (dN) and synonymous mutations per synonymous site (dS) was used to infer the direction and magnitude of natural selection acting on a protein encoding sequence. A dN/dS ratio lower than one implies purifying selection, whereas a ratio greater than one implies positive or fixing selection. The ratio was calculated using the Tamura-Nei method implemented in the MEGA software package that estimates the number of non-synonymous and synonymous changes that have occurred at each codon throughout evolution [33]. The average dN/dS ratio for the entire Env coding region was slightly less than one, suggesting that the entire *env* is under neutral selection. However, because different regions of a single gene can be exposed to different selective pressures, we analysed the dN/dS ratios over the entire length of the gene using a sliding window of 100 codons. Importantly, several regions in *env* were found to be under positive selection during virus cultivation in human cells (Figure 2A, left panel). These include segments of the *env* gene encoding the signal peptide, the receptor binding domain (RBD) at the N-terminus of the SU domain, the central part of SU and the N-terminal part of TM domain [20] (Figure 2A, right panel). Identification of a positive selection pressure for the RBD may be of particular interest as non-synonymous mutations in this region, which mediates interaction between the virus and receptor(s) on the cell surface, may alter virus tropism and facilitate infection of human cells. Interestingly, one of the nt substitutions leading to a non-conservative amino acid substitution (glutamic acid in place of glycine at position 42 of SU; G42E) in a five-amino-acid stretch of polar and hydrophobic residues, F_40_HGFR_44_, believed to constitute the receptor binding site (RBS) [20], was detected in all twelve MMTV(C3H)hp1 clones but not in MMTV(C3H) virus (Figure 2B and C). This particular polymorphism has previously been reported in the SU sequence from MMTV(RIII) virus adapted to the human breast cancer cell line MCF-7 (designated RIIIM) [20,34]. Additionally, one of the twelve MMTV(C3H)hp1 clones was also found to carry an additional non-synonymous mutation (arginine to lysine substitution at position 44 of SU; R44K) in the RBS of SU (Figure 2B). These results imply that the RBD of viruses replicating in human cells is under positive selection pressure and changes in this region may facilitate infection of human cells and in turn lead to faster replication kinetics. Other point mutations leading to amino acid substitutions were found outside the regions encoding the RBD and heparin binding domain (HBD). In contrast to the mutations described above, none of these polymorphisms were found in all twelve MMTV(C3H)hp1 clones.

Surprisingly, a high frequency of non-synonymous mutations was also identified for the Sag coding region (Figure 2A, left panel). This region also appears to be under a positive selection pressure. The reason for the observed phenomenon is not clear. As expected, the most conserved gene was the gene encoding the enzymatic components of the virus, *pol*. The dN/dS ratio for this region was close to one, suggesting that this segment of the virus was under neutral selection (Figure 2A, left panel).

### Virus replicating in human cells carries a human APOBEC3G signature of cytosine deamination

**Figure 3:**
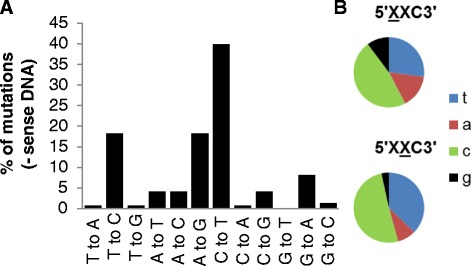
Virus replicating in human cells carries a human APOBEC3G signature of cytosine deamination. Nucleotide sequences obtained as described in Figure 3 were examined for the presence of hAPOBEC3G-mediated nucleotide changes on minus sense DNA. **(A)** All possible nucleotide transversions and transitions detected in the *pro* (a portion), *pol*, *env* and *sag* genes are expressed as a percentage of total number of nucleotide substitutions. **(B)** All C-to-T mutations found in the minus sense DNA strand were analysed for sequence context at the −1 and −2 nucleotide position.

Next, we analysed whether the MMTV(C3H)hp1 sequences carry a signature of the human APOBEC3G (hA3G), which is expressed in Hs578T cells [35]. The hA3G is an intrinsic immunity factor that catalyses deamination of deoxycitidine (C) to deoxyuridine (U) in the minus strand viral DNA (−ssDNA) during reverse transcription. (Subsequent synthesis of the plus strand from a deaminated minus strand template induces G to A mutations in newly generated plus strand viral DNA). We hypothesised that if the variants exhibiting faster replication kinetics resulted from the mutagenesis in human cells and not from selection of pre-existing quasispecies, they should contain hallmarks of hA3G-mediated mutagenesis (C to U transitions in the -ssDNA). Furthermore, analysis of sequence context of the deaminated deoxycitidines can be also used as an indirect method to confirm the human origin of infected cells. This is due to the fact that the hA3G and mouse APOBEC3 deaminases target deoxycytosine within different DNA sequence contexts (hA3G commonly targets the last C in a run of C’s on the -ssDNA, whereas mouse APOBEC3 prefers “TTC” targets [36-38]). We observed that about 40% of mutations could be attributed to APOBEC3 activity (Figure 3A; C to T substitutions in the –ssDNA). Additionally, the most commonly targeted cytosine was within the canonical 5′-CCC-3′ (−ssDNA) sequence context recognized by hA3G (Figure 3B). Importantly, the hA3G activity also likely accounted for the G42E substitution in the receptor binding sequence detected in all twelve MMTV(C3H)hp1 clones. The glycine codon GGG (CCC in -ssDNA) was mutated to GAG (CUC in –ssDNA) encoding for glutamic acid. Taken together, these data provide evidence supporting the concept that a large proportion of mutations in MMTV(C3H)hp1 proviral DNA can be attributed to hA3G-directed mutagenesis. Hallmarks of the hA3G-mediated cytosine deamination substantiate our original hypothesis that MMTV(C3H) is capable of productive infection of human cells. Additionally, the hA3G-mediated mutagenesis may, at least in part, explain the relatively long cultivation time required to detect infection of every human cell in culture.

### G42E alters the tropism of *egfp*-containing MMTV-based vectors

To find out whether the G42E mutation facilitates infection of human cells, the mutation was introduced into the MMTV(C3H) Env construct (pENV_C3H_). The wild-type and mutant ENVs were subsequently used to generate *egfp*-carrying MMTV recombinant virions, using an MMTV-based vector system analogous to the third generation of lentiviral vector production systems [39]. Construction of the vector system has been described elsewhere [39]. Briefly, replication incompetent vector particles were generated by cotransfection of HEK293T cells with an MMTV-based vector construct containing *egfp* gene, together with a packaging construct, an HIV-1 Rev expression construct and finally a plasmid expressing the WT (C3H) or mutant (G42E) envelope protein. As a control, we used MMTV particles pseudotyped with an amphotropic MLV 4070A Env. The filtered supernatant from transfected HEK293T cells was subsequently used to transduce both human (Hs578T) and mouse (NMuMG) mammary epithelial cells.

**Figure 4:**
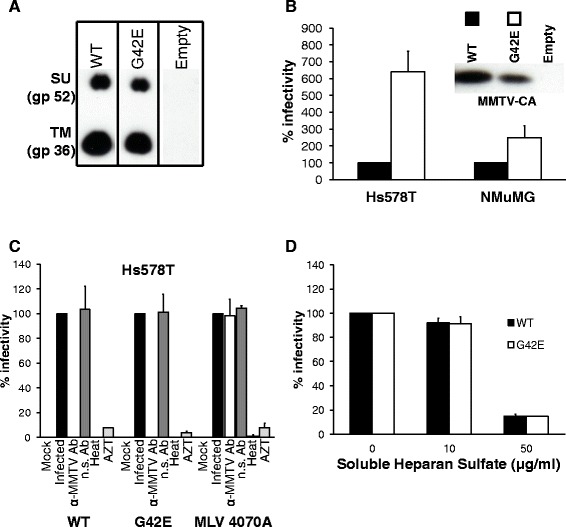
Mutation of a single amino acid in the RBS alters the tropism of *egfp*-containing MMTV-based vector. **(A)** MMTV Env carrying G42E mutation is expressed and cleaved into SU (gp52) and TM (gp36) subunits. Equivalent protein content (10 μg) of whole cell extracts from wild-type and mutant pENV_C3H_-transfected HEK293T cells were subjected to SDS-PAGE followed by Western blotting analysis with anti-gp52/36 (1:12000). **(B)** Target cells (Hs578T or NMuMG) were transduced with MMTV-based vector particles carrying wild-type or mutant MMTV(C3H) envelope. Transduction levels were made relative to the value obtained for MMTV-based vector particles carrying wild-type Env and expressed as a percentage of that control. The values shown are mean ± standard errors from three independent experiments. The inset shows a Western blot of 10 μl of each concentrated virus preparation probed with polyclonal anti-MMTV-CA antiserum (1:4000). **(C)** Hs578T cells were transduced with MMTV vectors carrying wild-type MMTV(C3H) Env, mutant MMTV(C3H) Env or MLV 4070A Env. Neutralization, heat inactivation and AZT treatment were performed as described in materials and methods. Transduction levels were made relative to the value obtained for the infected cells and expressed as percentage of that control. The values shown are mean ± standard errors from three independent experiments. **(D)** MMTV-based vector particles carrying either wild-type or mutant MMTV(C3H) Env were incubated with the indicated amount of heparan sulfate, and mixture was subsequently used to transduce Hs578T cells. Transduction levels were made relative to the value obtained in the absence of heparan sulfate and expressed as a percentage of that control. The values shown are mean ± standard errors from three independent experiments. Transduction was detected by flow cytometry (FCM) 3 days post infection. The number of eGFP expressing cells was counted using forward scatter vs. fluorescence intensity in the FL1 channel (eGFP) plots.

We first demonstrated that the mutant Env was efficiently processed. Total cell extracts from WT and mutant pENV_C3H_-transfected HEK293T cells were subjected to Western blot analysis with anti-gp52/36 to determine the levels of the viral proteins. The mutant Env was processed into mature SU and TM (Figure 4A), indicating that no gross changes in Env processing had occurred as a result of the sequence alterations.

The viruses were next tested for their ability to transduce Hs578T & NMuMG cells. When equal amounts of virus (quantified by western blot analysis of serially diluted concentrated virions using anti-MMTV CA antibody) were used for transduction of human cells, the G42E mutant exhibited approximately 6-fold increase in transduction efficiency compared to WT Env carrying virus (Figure 4B). In mouse cells, however, the same mutations caused only a modest (~2-fold) increase in transduction levels (Figure 4B). The infectivity of MMTV-based vectors carrying the mutant Env was, like the infectivity of vectors carrying the WT Env, completely blocked after pre-incubation with neutralizing goat anti-MMTV antibodies (Figure 4C). In contrast, pre-treatment of the virions with non-specific serum had no effect on the infectivity (Figure 4C). Importantly, no alteration in the infectivity was observed when the MLV 4070A Env-carrying pseudovirions were pre-incubated with the anti-MMTV antibodies (Figure 4C), ruling out nonspecific infection inhibition activity of the serum. Additionally, the infectivity of the WT and mutant Env-carrying MMTV virions was blocked by heat-inactivation (60°C, 10 minutes) and an inhibitor of reverse transcription, 3′-azido-3′-deoxythymidine (AZT; 37 μM) (Figure 4C), ruling out a non-specific mechanism of vector transfer/showing that vector transfer is due to an authentic, reverse transcriptase-dependent infection process.

Collectively, these data imply that G42E mutation is, at least in part, responsible for the adaptation of MMTV(C3H)hp1 to new host. Additionally, specific neutralization of vector infectivity demonstrates that mutant Env particles, like WT Env particles, rely on a specific interaction of the viral envelope with the cell surface receptor to mediate infection of human cells.

### G42E mutation does not enhance affinity to heparan sulfate

Viruses from different families (including retroviruses) that have undergone multiple passages in cell culture have been shown to exhibit a higher affinity for the glycosaminoglycan heparan sulfate (HS) [40-45]. To test whether the G42E mutation conferred an HS-binding phenotype, wild-type and mutant recombinant virus-containing supernatants were incubated in the presence or absence of increasing amounts HS and then used to transduce Hs578T cells. In agreement with previously published data [20], treatment of wild-type virus with soluble HS resulted in a dose-dependent decrease in infectious titers, demonstrating that binding to HS on the cell surface plays a role in MMTV infection (Figure 4D). Importantly, increasing amounts of HS caused a similar decrease in wild-type and mutant infectious titers, suggesting that the increased infectivity that accompanied the non-conservative G42E mutation was not due to enhanced interaction with proteoglycans (Figure 4D).

### G42E mutation enhances virus binding to human cells

**Figure 5:**
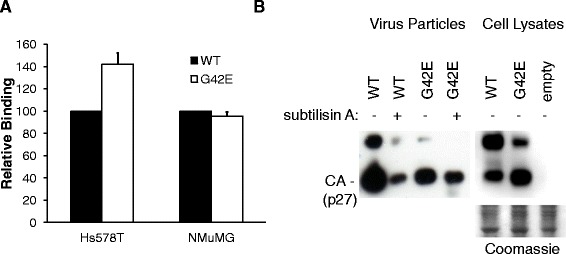
G42E enhances virus binding to human cells. **(A)** Equal amounts of MMTV-based vector particles carrying either wild-type or mutant (G42E) MMTV(C3H) Env were incubated on ice with either Hs578T or NMuMG cells. The cells were stained with anti-MMTV antiserum, followed by FITC-conjugated secondary antibodies and subjected to FCM. Dead cells were excluded by their forward scatter/side scatter properties. Binding levels were made relative to the value obtained for MMTV-based vector particles carrying wild-type Env and expressed as a percentage of the controls. The values shown are mean ± standard errors from three independent experiments. **(B)** Supernatants (left panel) from HEK293T cells cotransfected with packaging construct (pCMgpRRE17), an *egfp*-containing MMTV vector plasmid (pRRpCeGFPWPRE25), a Rev expression construct (pLP2) and either the wild-type or mutant MMTV(C3H) Env were harvested before and after subtilisin A treatment and virions pelleted by ultracentrifugation. 10 μl of the resuspended pellets was subjected toWestern blotting with anti-MMTV-CA antibodies. Cell lysates (right panel) of the virus producers before subtilisin A treatment were analysed by Western blotting. Equivalent protein loading was verified by Coomasie staining (bottom panel). The experiment was performed three times with similar results; a representative experiment is shown.

The increased infectivity of G42E mutant could be due to increased Env binding to its receptor or to alterations that affect subsequent steps in the infection pathway, such as membrane fusion. In an attempt to differentiate between the two possibilities virus-binding assays were performed. Hs578T and NMuMG cells were incubated with equal amounts of wild-type or mutant virions, stained with anti-MMTV antiserum and analysed by flow cytometry (FCM) to detect bound virus. Virus carrying the mutant MMTV Env bound human cells (Hs578T) more efficiently than virus carrying WT Env (Figure 5A). No difference in binding efficiency was observed for WT and mutant Env in NMuMG cells (Figure 5A).

To further corroborate the results, we analysed whether the virus carrying the mutant envelope is, due to tighter binding to the cell surface, less efficiently egressed from producer cells. Vectors, containing either the WT or mutant (G42E) Env, constitutively released from transfected cells and vectors trapped at the cell surface (released following subtilisin A treatment) were quantified by Western blot analysis with anti-MMTV-CA antibodies. The majority of the WT MMTV Env virions were released into the supernatant (Figure 5B, left panel, WT subtilisin A -) with relatively low amounts of virus trapped at the cell surface of producer cells (Figure 5B, left panel, WT subtilisin A +). In contrast, for mutant MMTV Env, a higher amount of virus was retained on the cell surface of producer cells (~4-fold more than amounts found for WT MMTV Env; Figure 5B, left panel, G42E subtilisin A +), which was also reflected by the lower amounts of constitutively released virus detected in the supernatant (Figure 5B, left panel, G42E subtilisin A -; and Figure 4B, inset).

Collectively, these data suggest that the non-conservative G42E mutation enhances the interaction between virus particles and their receptor(s) on the cell surface.

### Infection of human cells does not require expression of hTfR1

Next, we analysed whether the non-conservative G42E mutation, which alters the charge at RBS, allows MMTV to use hTfR1 for cell entry. Such a scenario would be reminiscent of what has been reported for New World clade B Arenaviruses, several of which have, through minor changes in the amino acid residues of the RBD, acquired compatibility with hTfR1, and are emerging into human populations through zoonotic transmission [26,46].

Chinese hamster ovary (CHO) cells, used for the identification of the MMTV receptor, were transfected with plasmids expressing the human and mouse TfR1, respectively. These cells were subsequently transduced with eGFP expressing MMTV-based vectors carrying either WT or mutant (G42E) Env. As controls, MMTV-based vectors pseudotyped with either the Junin (JUNV; pathogenic clade B arenavirus) or the Tacaribe (TCRV; non-pathogenic clade B arenavirus) glycoprotein (GP) were included. The GPs from pathogenic New World clade B arenaviruses use hTfR1 as a cellular receptor, whereas non-pathogenic strains do not use hTfR1 for cell entry [28-30,46]. mTfR1, on the other hand, plays no role in clade B arenavirus cell entry [28-30].

**Figure 6:**
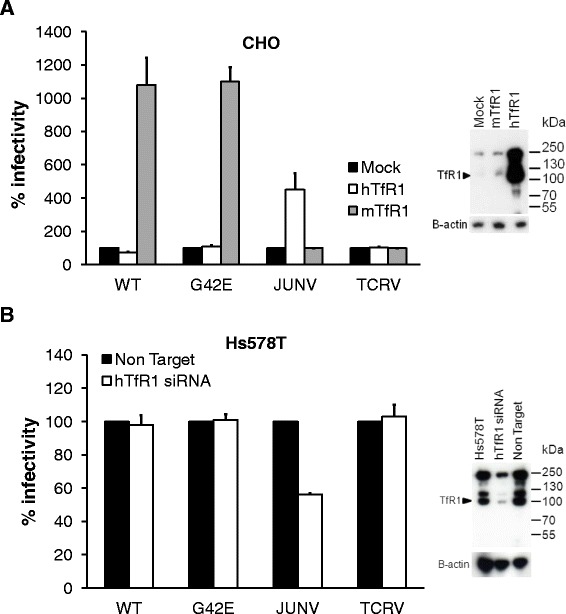
MMTV infection is not mediated through human transferrin receptor 1. **(A)** CHO cells transfected with mTfR1 or hTfR1expression plasmids were transduced with MMTV-based vector particles carrying the wild-type (WT) or mutant (G42E) MMTV(C3H) Env, or MMTV cores pseudotyped with clade B arenavirus GPs (Junin virus, JUNV; Tacaribe virus, TCRV). Expression of hTfR1 was confirmed by western blotting (inset); mTfR1 was not recognised by the α-TfR1 antibody (ab137944, Abcam). **(B)** Hs578T cells transfected with *hTFR1*-specific siRNA or non-targeting siRNA were transduced with MMTV-based vector particles carrying the wild-type (WT) or mutant (G42E) MMTV(C3H) Env, or MMTV cores pseudotyped with clade B arenavirus GPs. hTfR1 knock-down was confirmed by western blotting (inset). **(A & B)** Transduction of target cells was detected by FCM 3 days post infection. Transduction levels were made either relative to the value obtained for control siRNA **(A)** or relative to the value obtained for the mock-transfected cells **(B)** and expressed as percentage of those controls. The values shown are mean ± standard errors from three independent experiments.

As expected, the expression of hTfR1 in CHO cells resulted in a significant increase in the transduction levels of MMTV-based vectors pseudotyped with JUNV GP (~5-fold; Figure 6A) but did not affect the transduction rates of the vectors pseudotyped with TCRV GP (Figure 6A) [29,30,46]. Further, the addition of mTfR1 had no effect on the transduction efficiency of either JUNV or TCRV GP pseudotyped vectors (Figure 6A). In contrast to arenaviruses, mTfR1 expression caused a significant increase in the transduction levels of MMTV-based vectors carrying WT and G42E Env, respectively (>10 fold; Figure 6A). Importantly, the expression of hTfR1 had no effect on transduction efficiency (Figure 6A). Similar results were also obtained when wild-type MMTV(C3H) virus, harvested from cell culture fluids of an MMTV-induced mouse mammary tumour cell line (Mm5MT) was used (Additional file 1: Figure S1A). Also in this case only the presence of mTfR1 allowed efficient entry of the virus into CHO cells.

Next, we analysed whether a downmodulation of hTfR1 expression will have an effect on transduction efficiency. Hs578T cells were treated with an *hTFR1* mRNA-targeting siRNA prior to incubation with MMTV vectors carrying various Envs. As a control, cells transfected with non-targeting siRNA were used. Similar to previous reports, the depletion of hTfR1 in Hs578T cells resulted in approximately 2-fold reduction in the transduction levels of JUNV GP pseudotyped vectors, whereas the levels of TCRV GP pseudotyped vectors remained unaffected (Figure 6B) [30]. Importantly, the transduction efficiency of MMTV Env-carrying vectors under conditions of reduced endogenous hTfR1 expression remained comparable to that obtained with cells expressing hTfR1. Similar results were observed for both the WT and mutant Env (Figure 6B). The infectivity of wild-type MMTV(C3H) virus, harvested from Mm5MT cells, was also unaffected by hTfR1 knock-down (Additional file 1: Figure S1B).

Taken together, these results show that mTfR1 can serve as receptor mediating entry of wild-type virus and vectors containing the wild-type as well as G42E Env. Furthermore, the presented evidence suggests that MMTV variants carrying G42E mutation, although they exhibit greater infectivity in human cells, do not use hTfR1 to mediate their entry into human cells.

### MMTV infects hTfR1 knock-out Hs578T cells

Knock-down of *hTFR1* did not result in complete elimination of the hTfR1 protein expression. Therefore, it may be possible that residual levels of hTfR1 may affect the MMTV entry process. For example, remaining hTfR1 may be sufficient to initiate a low level of virus entry. Alternatively, as MMTV binds to hTfR1 [6], the presence of the protein on the cell surface may trap the virus and/or hinder its interaction with other molecules on the cell surface. To provide a definitive answer as to whether hTfR1 has any role to play in MMTV infection, we knocked out *hTFR1* from Hs578T cells, using CRISPR (Clustered Regularly Interspaced Short Palindromic Repeats Type II)/Cas9 [47,48]. Specifically, we used the lentiCRISPR vector production system, which enables simultaneous delivery of a mammalian codon-optimised Cas9 nuclease, a single guide RNA (sgRNA) and a puromycin selection marker into target cells [49].

To test the efficacy of the gene knock-out by lentiCRISPR transduction, a sgRNA targeting *egfp* was cloned into the LentiCRISPR plasmid. The LentiCRISPR plasmid was transfected into HEK293T cells together with a packaging vector, an HIV rev expression construct and a vector encoding the vesicular stomatitis virus (VSV-G) Env to generate replication-defective lentiviral vectors for transduction. The filtered supernatants from transfected HEK293T cells were subsequently used to transduce Hs578T cells expressing EGFP. Following 11 days selection with puromycin, lentiCRISPR abolished EGFP fluorescence in the majority (~98%) of cells, as determined by flow cytometry analysis (data not shown).

**Figure 7:**
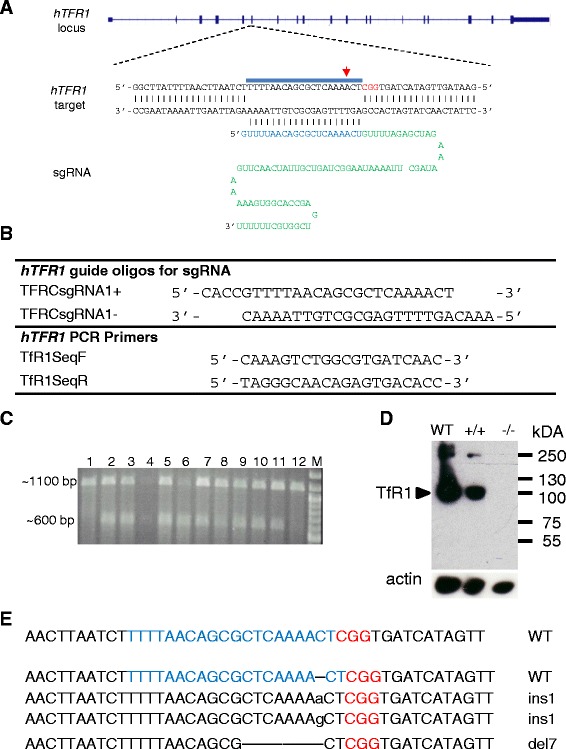
Generation of hTfR1 knock-out cells using CRISPR/Cas9. **(A)** TFR1 locus on the human chromosome 3 targeted by CRISPR/Cas9. A sgRNA consisting of 20-nt guide sequence (blue) and a scaffold (green). The guide sequence anneals with the DNA target (blue bar) upstream of PAM (red). A red arrow points to the expected cleavage site. **(B)** Oligonucleotide sequences used for the generation and characterisation of Hs578TΔhTfR1 cells. **(C)** Detection of mutations in Hs578T clones generated by transduction of lentiCRISPR vector containing sgRNA targeting *hTFR1*and Cas9 nuclease. PCR products (1100 bp) containing the target site were denatured, re-annealed and probed with T7 endonuclease that recognizes non-perfectly matched DNA. A cleavage product of ~600 bp indicates successful NHEJ-mediated mutation in at least one copy of *hTFR1* (lanes 2–11). Analysis of 12 clones is shown; (the amount of PCR product loaded in lane 4 was lower than in other lanes). **(D)** Western blot analysis of the *hTFR1* knock-out Hs578T clone (−/−) together with controls including parental cells (WT) and a clone containing at least one copy of non-mutated *hTFR1*(+/+). Cellular lysates were also probed for actin to ensure equal loading (bottom panel). **(E)** Sequence analysis of the *hTFR1* target site of the clone bearing mutations in all three copies of the target site in Hs578T cells.

**Figure 8:**
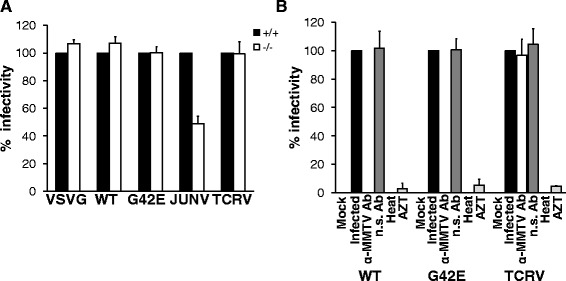
*hTFR1* knock-out Hs578T cells are susceptible to MMTV infection. **(A & B)** Target cells were transduced with MMTV-based vectors carrying wild-type MMTV(C3H) Env, G42E MMTV(C3H) Env, JUNV GP or TCRV GP. Neutralization, heat inactivation and AZT treatment were performed as described in materials and methods. Transduction of target cells was detected by FCM 3 days post infection The number of eGFP-expressing cells was counted using forward scatter vs. fluorescence intensity in the FL1 channel (eGFP) plots. **(A)** Transduction levels were made relative to the value obtained for the infected cells expressing detectable levels of hTfR1 and expressed as percentage of that control. **(B)** Transduction levels were made relative to the value obtained for the infected cells and expressed as percentage of that control. The values shown are mean ± standard errors from three independent experiments.

For targeted *hTFR1* knock-out, sgRNA targeting a sequence within the 6th exon of *hTFR1* was cloned into the LentiCRISPR plasmid and used for transduction of Hs578T cells (Figure 7A and B). Transduced cells were cultured in the presence of puromycin for a total of 14 days after which individual clones were isolated and expanded. A total of 43 clones were used for further analysis. First, we assayed the genomic mutation status of the *hTFR1* locus in the individual clones. The CRISPR-Cas9 system induces double-strand DNA breaks that are repaired mainly by error-prone non-homologous end joining (NHEJ). Thirty three percent (14 out of 43) of the clones bore mutations at the *hTFR1* locus, as determined by a T7 endonuclease I (T7EI) assay and DNA sequencing (Figure 7C). Of the 14 clones, one contained mutations in all three copies of the *hTFR1* locus [Hs578T cells carry two copies of chromosome 3 as well a derivative of chromosome 3: der(3)t(3;15)(q10;p10); *hTFR1* gene located on Chr3q29], as determined by DNA sequencing (Figure 7E). These mutations led to premature stop codons, which effectively disrupted the open reading frame (ORF) of *hTFR1*, yielding truncated protein products (Additional file 2: Figure S2). Successful hTfR1 knock-out was confirmed by western blotting of cell lysates (Figure 7D). Next, these Hs578TΔhTfR1 cells were transduced with the panel of MMTV-based vectors carrying WT MMTV(C3H) Env, mutant (G42E) Env, JUNV GP or TCRV GP. As a control, one of the clones that carried no mutations at the *hTFR1* and showed similar levels of hTfR1 expression to parental Hs578T cells was used. As expected, transduction efficiency of JUNV GP-pseudotyped vectors was compromised in Hs578TΔhTfR1 cells compared to cells expressing hTfR1 (Figure 8A). Further, TCRV GP vector transduction rates were unaffected by hTfR1 expression (Figure 8A) [29,30]. In contrast, MMTV-based vectors carrying either WT or mutant MMTV(C3H) Env transduced Hs578TΔhTfR1 cells with similar efficiency to cells expressing hTfR1 (Figure 8A).

The ability of MMTV-based vectors carrying either WT or mutant MMTV(C3H) Env to transduce Hs578TΔhTfR1 cells was completely blocked after pre-incubating with neutralizing goat anti-MMTV antibodies (Figure 8B). In contrast, pre-treatment of the virions with non-specific serum had no effect on the ability of these vectors to infect Hs578TΔhTfR1 cells (Figure 8B). Importantly, TCRV GP pseudotyped vectors, pre-incubated with the anti-MMTV antibodies, were still able to transduce Hs578TΔhTfR1 cells (Figure 8B), ruling out nonspecific infection inhibition activity of the serum. These results suggest that MMTV-mediated infection of Hs578TΔhTfR1 cells relies on a specific interaction of the viral envelope with a cell surface receptor, other than hTfR1. Furthermore, the incubation of supernatants at 60°C for 10 minutes, prior to transduction of hTfR1 knock-out cells, abrogated the infectivity of vectors regardless of the Env used (Figure 8B), showing that transmission of vector DNA is temperature sensitive, as expected for vector transduction rather than for other means of DNA transfer. Finally, the addition of AZT (37 μM) to the culture medium of the transduced Hs578TΔhTfR1 cells inhibited transduction (Figure 8B), further demonstrating that the presence of vector DNA in the transduced cells resulted from the reverse transcription-dependent process as expected for vector transduction.

Collectively, the knock-out experiments further support the concept that MMTV is capable of infecting cells in the absence of TfR1. The identified mutation in the receptor binding site of MMTV Env that emerged as a result of positive selection in cells lacking the primary receptor, mTfR1, further amplified this phenotype. Therefore, we propose that a molecule(s) on the cell surface, other than TfR1, is capable of mediating entry of MMTV into cells and thus serves as an alternative receptor/coreceptor.

## Discussion

The data presented here demonstrate that MMTV(C3H) released from persistently infected human cells [MMTV(C3H)hp1] exhibits a markedly faster replication kinetics in human cells than the parental virus. Specifically, MMTV(C3H)hp1 required four-times less time (21 days) to infect every cell in cell culture, even-though infectious doses equivalent to those used with viruses produced from murine cells were used. Several mechanisms may explain the observed variation in replication kinetics: i) differences in virus preparations, such as percentage of defective virions; ii) pseudotyping with human genome-encoded envelope protein(s); iii) recombination with human cellular DNA or human endogenous proviral sequences; iv) selection of a pre-existing quasispecies in the wild-type MMTV population and v) random mutation event(s) followed by selection of variants exhibiting faster replication kinetics in human cells.

We believe that the first two mechanisms can be readily ruled out. This conviction is based on the observation that MMTV(C3H)hp1 virions contained surface antigen determinants identical or very similar to those found on MMTVs released from murine cells, as judged from the complete neutralization of MMTV(C3H)hp1 infectivity with the MMTV-specific antibodies. Similarly, real time RT-PCR results, combined with single round infection experiments, did not reveal significantly greater levels of defective viruses in virus preparations generated in murine and human cells, respectively. Furthermore, MMTV(C3H)hp1 did not contain sequences from other viruses or from the human genome, as determined by sequences analysis. Instead, the MMTV(C3H)hp1 sequences (complete genomes with the exception of the Gag-coding region containing a sequence precluding cloning and growth in *E. coli*) originated from MMTV(C3H). Thus, recombination with sequences of human origin does also not account for the observed variation in the speed of virus replication. Finally, upon further analysis, MMTV(C3H)hp1 was found to carry an hA3G specific signature of cytosine deamination. Hallmarks of the hA3G-mediated cytosine deamination further substantiate our original hypothesis that MMTV(C3H) is capable of productive infection of human cells. Additionally, the hA3G-mediated mutagenesis may, at least in part, also explain the relatively long cultivation time required to detect infection of every human cell in culture.

Next, we analysed whether a selection pressure shaped genetic variation during replication in human cells. We found that some regions within the MMTV(C3H)hp1 genome, including the segments of the *env* gene encoding the RBD at the N-terminus of the SU domain, the central part of SU and the N-terminal part of TM domain, were under strong positive (fixation) selection pressure. Most of the point mutations identified in the SU subunit-encoding portion of *env* were unique to the MMTV(C3H)hp1 sequences and were not found in other strains of MMTV. Additionally, the bulk of the polymorphisms were unique to each individual proviral plasmid clone. Several non-synonymous mutations were found in the RBD. Some of these mutations were situated directly in a five-amino-acid stretch of polar and hydrophobic residues, F_40_HGFR_44_, believed to constitute the RBS [20]. These mutations in the RBS may thus alter MMTV SU tropism and allow MMTV to adapt to a new host range.

Of note, all MMTV(C3H)hp1 viruses, in contrast to their parental virus MMTV(C3H), carried a glutamic acid substituted for glycine at position 42 of SU (G42E) (Figure 2B). This residue has been shown to map to the outer surface of the SU domain and to occupy a position within a concave surface [20], consistent with a role in receptor binding/interaction. The non-conservative alteration from a small non-charged amino acid to a negatively charged polar residue is expected to impose both electrostatic and steric effects on the RBS (Figure 2C). This mutation may thus alter virus receptor use, giving rise to an adapted virus capable of infecting heterologous cells with increased efficiency. In HIV, mutations that alter the charge in and around the predicted CD4-binding site in gp120 give rise to variants that enter cells in a CD4-independent manner, which enables the virus to expand its target cell range [50]. Additionally, charge has long been recognized to differ between R5-tropic and X4-tropic V3 loop sequences [51]. Mutations that alter the V3 loop charge have been shown to play an important role in determining virus tropism i.e. which coreceptor is used to mediate cell entry [52].

The introduction of charged glutamic acid in place of glycine in the WT MMTV(C3H) Env greatly increased the efficiency with which the MMTV recombinant virions transduced human cells. In contrast, G42E substitution caused only a modest alteration in transduction levels of mouse cells. Furthermore, MMTV recombinant virions carrying glutamic acid at position 42 bound human cells more efficiently than virions carrying WT Env. No difference in binding was, however, observed for wild-type and mutant virions in mouse cells. These data imply that MMTV(C3H) produced from murine cells is able to adapt to a new host range, at least in part, by shaping the receptor binding domain to optimize the interaction with its receptor(s).

Studies carried out with MMTV(C3H) Env-pseudotyped MLV viruses showed that mTfR1 is used by MMTV to initiate infection of mouse cells [5]. The human ortholog (hTfR1), however, was reported as a molecule incapable of mediating MLV/MMTV(C3H) entry, even though it bound pseudovirus efficiently [6]. Therefore, we initially hypothesized that MMTV(C3H) that replicates in human cells adapts, by mutating RBS, to enable utilization of hTfR1 for cell entry. Such a scenario would be reminiscent of what has been reported for another group of viruses that use TfR1 as their primary cellular receptor - New World clade B Arenaviruses. These viruses are endemic to rodent populations found in the Americas and each virus has evolved compatibility with the specific TfR1 ortholog encoded by its respective host species [18,28-30]. Several of these viruses have, however, through changes in the amino acid residues of the RBD, acquired compatibility with hTfR1, which allows more efficient infection of human cells and enables the zoonotic transmission to humans [26,46].

However, in the case of MMTV, the G42E mutation that resulted from adaptation to human cells did not confer hTfR1 dependence. This belief is based on the following evidence. First, the transient expression of hTfR1 in rodent (CHO) cells did not increase susceptibility to MMTV-based vectors carrying the mutant Env relative to vectors carrying WT Env. Second, human (Hs578T) cells, in which the TfR1 expression has been down-modulated using *hTFR1*-targeted siRNA did not exhibit decreased susceptibility to MMTV-based vectors carrying the mutant Env relative to vectors carrying the WT Env. Third, MMTV-based vectors carrying mutant MMTV(C3H) Env transduced *hTFR1* knock-out human Hs578T cells with efficiency similar to cells expressing hTfR1. Additionally, when the binding rates of WT and mutant MMTV Env-proteins (expressed in 293 T cells) to a His-tagged recombinant-hTfR1 protein (immobilized on a His-Ab coated biosensor) were assessed using the BLItz System (ForteBio), the kinetic characteristics of MMTV Env with and without the G42E mutation showed no detectable differences in the interaction with hTfR1 (data not shown).

Even-though the G42E mutation in the RBS does not adapt MMTV to using hTfR1 for cell entry, it clearly shapes the receptor binding domain to optimize the interaction with its (as yet unknown) receptor(s) as evidenced by an enhanced infectivity of virions carrying the G42E Env. This interaction could be neutralized by specific anti-MMTV serum further supporting the concept that a specific interaction of viral envelope with a cell surface receptor, other than hTfR1, mediates entry into human cells.

The concept of an alternate receptor is further supported by other evidence. This includes the observation that feline TfR1, derived from the most susceptible cell line, CrFK, does not make cells susceptible to MMTV infection [53]. Thus, feline cells must express another molecule, different from TfR1 that enables the virus to enter the cytoplasm. Further evidence comes from studies of cell entry of the non-pathogenic New World arenaviruses. They typically use TfR1 orthologs of their host species as receptor molecule. However, like MMTV, they have been reported to relatively efficiently enter human cells in an TfR1 independent manner using so far unidentified alterantive receptor(s) [29,30]. Additionally, the usage of more than one cell surface molecule as receptor is not rare and it has been reported for a number of viruses including SARS coronavisus and enterovirus 71 [54,55]. Some viruses seem to exhibit an even more complex receptor dependency that involves engagement with multiple cell surface components. An example is hepatitis C virus (HCV), which in addition to attachment factors such as HS and L-SIGN also requires the co-expression of four proteins (CD81, SR-B1, claudin-1 and occludin) for entry into cells (reviewed in [56]). Another variation is the use of both a primary and a secondary/co-receptor for cell entry. HIV-1 is a prime example of such a process, since it requires the co-expression of co-receptors (CCR5 or CXCR4), in addition to the primary receptor CD4, for cell entry (reviewed in [57]). Finally, some viruses use less specific interactions with attachment factors to bind to the cell surface before interacting with the true receptor, such as the binding of HIV-1 to syndecans, which enhances virus entry into macrophages [58]. In the case of MMTV, an alternative receptor may substitute for hTfR1, function in conjuction with TfR1 in a manner similar to the HCV multiple cell surface component dependency, or function down-stream of TfR1 in manner analogous to the HIV1 co-receptors. Work to identify the attachment receptor used by MMTV to enter human cells and initiate infection is currently ongoing.

## Conclusion

The replication of MMTV(C3H) in human cells led to the adaptation of the virus to its new host by shaping the receptor binding domain to optimize the interaction with its receptor(s). Our data explain the mechanism enabling MMTV to form ‘host-range variants’ in non-murine cells that has been known for a long time, the basis of which remained obscure. The receptor mediating infection of human cells remains to be identified as we ruled out engagement of hTfR1 on the entry process. The identified mutation (G42E) in the receptor binding site that has emerged as a result of positive selection pressure in human cells may serve as a marker of MMTV replication in human cells.

## Methods

### Cell culture

HEK293T (ATCC CRL-3216), Hs578T (ATCC HTB-126), NMuMG (ATCC CRL-1636), CHO-K1 (ATCC CCL-61) and Mm5MT (ATCC CRL-1637) cells were maintained in DMEM medium supplemented with 10% fetal bovine serum (FBS). All cultured cells were maintained at 37°C in humidified air containing 5% CO_2_.

### Generation of Hs578T cells expressing EGFP

To generate an Hs578T-EGFP stable cell line, expressing the green fluorescent reporter protein (EGFP) and the neomycin resistance marker, Hs578T cells (plated at a density of 2.4 × 10^5^ cells per well of a six-well plate one day before transfection) were transfected with 1 μg of pCMV-EGFP vector (Clontech) using the TurboFect transfection reagent (Thermo Scientific). Twenty-four hours post-transfection, normal growth medium was replaced with medium containing G418 (400 μg/ml; Invitrogen) and the cells were cultured for two weeks. The expression of EGFP in G418-resistent cells was analysed by UV microscopy and FCM.

### Generation of *hTFR1* knock-out HS578T cells

*hTFR1* was knocked out in Hs578T cells using the lentiCRISPR vector system as described before [49]. Oligonucleotide primers specific for either *hTFR1* (Figure 7A & B) or *egfp* were designed according to Shalem *et al*. [49]. The gene specific oligos were phosphorylated at the 5′ end using T4 polynucleotide kinase (PNK), annealed together giving rise to a double stranded insert carrying cohesive ends compatible with BsmBI-generated restriction sites. Reactions were carried out in a total volume of 10 μl containing 100 μM of each oligo, 1x T4 Ligation Buffer (NEB) and 5 units T4 PNK (NEB). After the first incubation at 37°C for 30 minutes, the oligos were denaturated at 95°C for 5 min and annealed together by gradually ramping down temperature (−5°C/min) to 25°C. The lentiCRISPR vector was digested with BsmBI (NEB) to remove a spacer sequence and ligated with the double stranded insert. To produce lentivirus vector, 7.5 x 10^5^ HEK293T cells (6-well plate) were co-transfected with 1 μg lentiCRISPR plasmid, 0.8 μg packaging construct (pLP1, Invirogen), 0.6 μg Rev expression construct (pLP2, Invitorgen) and 0.3 μg VSV-G Env-encoding plasmid (pHCMV-G). Transfections were performed using the calcium phosphate method. Thirty-six hours post-transfection, the virus-containing supernatants were harvested, filtered (0.45 μm, Sarstedt) and 4 serial dilutions (undiluted, 1:10, 1:100 and 1:1000) prepared. For transduction, target cells (Hs578T or Hs578T-EGFP) were seeded at a density of 5 x 10^4^ cells per well (6-well plate), one day before transduction. The increasing dilutions of the filtered virus supernatant was added to cells together with polybrene (8 μg/ml) and replaced with fresh medium after 2 hours. Puromycin (0.3 μg/mL) was added to the cells 16 hours post transduction. Hs578T-EGFP cells transduced with lentiCRISPR-based vector particles carrying sgRNA targeting *egfp* were maintained for 11 days and analysed by UV microscopy and FCM. Hs578T cells transduced with lentiCRISPR-based vector particles carrying sgRNA targeting *hTFR1* were maintained for 14 days in the puromycin-containing medium. Then, single clones were expanded, genomic DNA extracted and analysed by a T7 endonuclease I assay followed by DNA sequencing. For the T7 endonuclease assay the primers listed in Figure 8A were used to generate an 1100 bp PCR product encompassing the target site. Cycling conditions consisted of 94°C for 2 min, followed by 35 cycles of 94°C for 30 s, 58°C for 30 s, 72°C for 40 s, and a final extension of 7 min at 72°C. The PCR products were column purified and 500 ng of DNA per sample were subjected to the following hybridisation reaction: 95°C for 5 min, ramped down to 85°C at −2°C /s and ramped down further to 25°C at −0.1°C/s. The products were subsequently digested with 1 unit of T7 endonuclease (NEB) in a 10 μl reaction volume for 30 min at 37°C. The mixture was then resolved on a 2% agarose gel. The amplicons carrying non-perfectly matched DNA (digested with T7 endonuclease) were cloned into the pCR2.1 plasmid [TA cloning kit (Invitrogen)] and at least six clones analysed by sequencing.

### Pseudovirus production and transduction

*egfp*-containing MMTV recombinant virions carrying one of the following envelope proteins MMTV Env, amphotropic MLV Env, Junin GP or Tacaribe GP were prepared in HEK293T cells as described before [39] and used for transduction of target cells. Briefly, 7.5 x 10^5^ cells (6-well plate) were co-transfected with a packaging construct (pCMgpRRE17; 0.8 μg), an MMTV vector plasmid (pRRpCeGFPWPRE25; 1 μg), a Rev expression construct (pLP2; 0.6 μg) and a construct expressing either MMTV(C3H)Env (0.3 μg), amphotropic MLVEnv (pAlf; 0.3 μg) [59], a pathogenic clade B arenavirus GP (JUNV; 0.3 μg) [30] or a non-pathogenic clade B arenavirus GP (TCRV; 0.3 μg) [30]. Thirty-six hours post-transfection, the virus-containing supernatants were harvested, filtered (0.45 μm, Sarstedt) and used for transduction or stored at −80°C. Target cells for transduction were seeded at a density of 5 x 10^4^ cells per well (6-well plate), one day before transduction. The vector containing supernatant was applied onto cells together with polybrene (8 μg/ml) and replaced with fresh medium after 2 hours. Three days post-transduction, cells were analysed by FCM.

### Infections with MMTV(C3H) virus from Mm5MT

Filtered (0.45 μm, Sarstedt) cell culture fluids from dexamethasone- stimulated (DEX; 10^−6^ M; Sigma-Aldrich) Mm5MT cells (producing infectious MMTV(C3H) particles) (6.5 x 10^4^ RNA copies) were used to infect target cells seeded at a concentration of 5 x 10^4^ cells per well (6-well plate), one day before infection. The supernatant was added to cells together with polybrene (8 μg/ml) and replaced with fresh medium after 2 hours. For single round infection experiments, infected cells were cultured for one week and analysed by PCR as described below. For time-course experiments, infected Hs578T cells were further cultivated in cell culture medium either supplemented or not with DEX (10^−6^ M) for 7–16 weeks. Semi-quantitative LTR-*gag*-specific PCR and qPCR was used to quantify proviral DNA loads during the course of the experiment. To directly demonstrate the production of infectious MMTV particles from persistently infected Hs578T cells [MMTV(C3H)hp1], supernatants from the infected cells cultured with DEX for six weeks were filtered and used to infect naive, uninfected target cells. The LTR-*gag* PCR was employed to monitor the presence of proviral sequences in the second round infected cells. For all infection experiments normalized virus titers (6.5 x 10^4^ RNA copies) were used to infect target cells seeded in 6-well plates.

### Neutralization of viral infectivity, heat inactivation and AZT treatment

In infection neutralization experiments, vector/virus harvested from producer cells was incubated with polyclonal goat anti-MMTV antiserum (1:500; kindly provided by S. Ross) or non-specific serum (1:500) on ice for 30 min before infection. In heat inactivation experiments, virus/vector was incubated at 60°C for 10 min prior to infection. In 3′-azido-3′-deoxythymidine (AZT) treatment experiments, AZT (37 μM) was added to naive cells together with the virus inoculum. After 2 hours incubation at 37°C, the supernatant was replaced with fresh medium containing AZT (37 μM).

### Heparan sulfate competition assay

MMTV-based vector particles carrying either WT Env or the G42E mutant Env were incubated with the indicated amount of heparan sulfate (Sigma-Aldrich) at 37°C for 1 h. Polybrene (8 μg/ml) was added, and this mixture used to infect Hs578T cells. After incubation for 2 h at 37°C, the cells were re-fed with fresh medium. Three day post infection, cells were analysed for infection by FCM.

### Virus binding assay

Virus binding assay was performed as previously described [20]. Briefly, MMTV recombinant virions carrying either WT Env or G42E Env were generated as described above. The vector particles were concentrated by ultracentrifugation at 20,000 rpm for 2 h in an SW40 rotor, resuspended in 200 μl of phosphate-buffered saline (PBS, pH 7.4) containing 2% FBS and 1 mM EDTA (PBS-FE). Relative quantities of WT Env- and G 42E Env-carrying virions in the preparations were determined by Western blotting using anti-MMTV CA antibody and serially diluted virus stocks. Single-cell suspensions of Hs758T or NMuMG cells were prepared by incubation of the cells with PBS-FE for 15 min, followed by vigorous pipetting. Then, equal amounts of virus particles containing either WT Env or G42E Env (normalized based on Western blot results) was incubated with 2.5 × 10^5^ cells in the presence of Polybrene (8 μg/ml) at 4°C for 1 h. The cells were washed three times and resuspended in 100 μl of ice-cold PBS containing 1% FBS and 0.2% sodium azide. Then, 100 μl of a 1:100 dilution of goat anti-MMTV polyclonal antiserum was added and incubated for additional 30 min at 4°C. The cells were washed three times and incubated with 100 μl of fluorescein isothiocyanate (FITC)-conjugated rabbit anti-goat immunoglobulin antibody. The cells were washed three times, and analysed by FCM.

### Subtilisin A treatment

Virions that remain attached to the cell surface of the MMTV vector producing cells were quantified following subtilisin A treatment as previously described [60]. Briefly, transfected HEK293T cells (6 cm dishes) producing recombinant virions carrying either WT Env or G42E Env were washed twice with PBS, once with subtilisin A buffer (10 mM Tris pH 8.0, 1 mM CaCl_2_, 150 mM NaCl) and treated with 500 μl of 1 mg/ml subtilisin A (Sigma-Aldrich) for 3 minutes at room temperature. Subtilisin A treatment was stopped by adding DMEM containing 10% FCS, 5 mM PMSF and 20 mM EGTA. Virions released after subtilisin A treatment as well as virions constitutively released from cells were concentrated by ultracentrifugation (20,000 rpm for 2 h in an SW40 rotor). The virus pellets were resuspended in 50 μl of lysis buffer (140 mM NaCl, 20 mM Tris pH 8.0, 1% Triton X-100) and 10 μl of the resuspended pellets was subjected to SDS-PAGE followed by Western blotting analysis with anti-MMTV-CA antibodies.

### Western Blot

Equivalent protein content of whole cell lysates (measured using DC Protein Assay; Bio-Rad) or equal volumes of concentrated MMTV vectors were resolved using a 12% polyacrylamide gel and transferred to Hybond-P membranes (GE healthcare). The membranes were probed with the following antibodies: for detection of TfR1 – the rabbit anti-transferrin receptor antibody (Abcam: ab84036; dilution 1:1000), for detection of beta-actin – the rabbit anti-actin antibody (Sigma-Aldrich: A2066; dilution: 1:100), for detection of MMTV capsid protein – the rabbit anti-MMTV-CA antibody (Michael Sakalian; dilution: 1:4000), for detection of MMTV SU and TM subunits of MMTV Env – the rabbit anti-gp52/36 (Janet Butel; dilution 1:12000). The horseradish peroxidise-konjugated swine anti-rabbit IgG (DAKO: P0399; dilution 1:10000) secondary antibody was used as secondary antibody followed by incubation with the ECL Prime Western Blotting Detection Reagent Kit (GE Healthcare). Luminescence signals were detected by exposure to Amersham hyperfilm™ (GE healthcare) and/or by scanning using LI-COR C-DIGIT blot scanner, which also allows quantification of signals.

### Semi-quantitative PCR

Genomic DNA (gDNA) was isolated from cells using the QIAgen DNeasy Blood and Tissue Kit (Qiagen). The presence of MMTV proviral DNA in infected cells was detected by PCR using primers designed to amplify a 717 bp long segment spanning the MMTV LTR-*gag* region (935 F: 5′-AAG ACG ACA TGA AAC AAC AG-3′; 1637R: 5′-CCC AGT TCC AAT GGC TCA CCG TAA-3′). 200 ng of gDNA was used as a template for amplification. Cycling conditions consisted of initial denaturation at 94°C for 2 min, followed by 35 cycles of 94°C for 30 s, 58°C for 1 min, 72°C for 40 s, and a final extension at 72°C for 7 min. Equal loading of each PCR reaction was controlled using a GAPDH-specific primer pair (GAPDH F: 5′-ATG GCT CCT GCA CCA CCA AC-3′; GAPDH R: 5′-CGC CTG CTT CAC CAC CTT CT-3′) in a PCR reaction (only 25 cycles) carried out with the identical sample amounts as in the MMTV-specific PCR.

### Quantitative PCR

Proviral loads in infected cells were quantified by a Real-time TaqMan PCR using primers targeting the 5′ end of the MMTV *env*. 100 ng of gDNA was amplified using the following set of primers: MMTV 01 F: 5′-GGA AAG TCC GGA GGA TGA ATC TA-3′, MMTV 02R: 5′-CTC CGC TTC GGA GAT TAA CG-3′ and a TaqMan probe: 5′-FAM-CAT CAA AGA GAA GAC GGC TTG GCA ACA TC-TAMRA-3′. In each TaqMan experiment, a standard was run consisting of a serially diluted plasmid carrying a molecular clone of MMTV (pGR102) [61]. Cycling conditions consisted of 95°C for 2 min followed by 40 cycles of 95°C for 30 s and 60°C for 1 min. The threshold cycle (*C*_τ_) was measured for each well and a standard curve was plotted using the threshold cycle values of the serially diluted plasmid DNA. Equal loading of PCR reactions was verified using a TaqMan Real-time PCR specific for the human *APOB* gene using the following primers and a TaqMan probe: hAPOB F: 5′-TTC TTA CCA CAC ATC TCT TGA TTC TCT T-3′, hAPOB R: 5′-GGA CTT CAC TGG ACA AGG TCA TAC T-3′ and TaqMan probe: 5′-FAM-CAC TCG TCC AGG TGC GAA GCA GAC T-3′. Identical PCR conditions as outlined above were employed.

### Amplification of viral RNA or proviral DNA followed by sequencing

gDNA was isolated from Hs578T cells two weeks after infection with MMTV(C3H)hp1 virus harvested from persistently infected Hs578T cells. Viral RNA was extracted from supernatants of Mm5MT cells. TRI Reagent (Sigma-Aldrich) was used for extractions. For cDNA synthesis, RNA treated with DNAse I (TURBO DNA-free kit; Ambion) was reverse transcribed using a primer 9877_R (5′-TCA GCA CTC TTT TAT ATC TTG G-3′) and Superscript II reverse transcriptase (Invitrogen). gDNA and cDNA were subsequently used for a long-template PCR (Expand Long Template PCR system; Roche), using primers 3722_F (5′-CGG *GGT ACC* GGC TCA GAA GGC TTC GGA TC-3′) and 9761_R (5′-ATA AGA AT*G CGG CCG C*GG CTC AGA AGG CTT CGG ATC-3′), carrying Acc65I and NotI restriction sites (italics), respectively. The resulting PCR fragments were cloned into the corresponding sites of the pcDNA3 vector (Invitrogen) and sequenced using overlapping primers available from the authors upon request.

### Knock-down of *hTFR1* by small interfering RNA

Hs578T cells (plated at a density of 3 × 10^5^ cells per well of a six-well plate, one day before transfection) were transfected overnight with human TFRC SMARTpool (25 nM, Dharmacon) or non-targeting pool #2 (Dharmacon) siRNA using DharmaFECT (Thermo Scientific). Forty-eight hours post-transfection, the cells were either infected with wild-type MMTV(C3H) virus or transduced with *egfp*-containing MMTV-based vector particles carrying the WT or mutant (G42E) MMTV(C3H) Env. Infection of target cells with MMTV(C3H) virus was assessed by the LTR-*gag* PCR one week after infection. Transduction of target cells with *egfp*-carrying MMTV vectors was assessed by FCM 72 hours post-transduction.

### TFR1 overexpression

CHO and Hs578T cells (plated at a density of 2 × 10^5^ cells per well of a six-well plate, one day before transfection) were transfected with 4 μg of a mTfR1 expression vector (pcDNA3.1-mTfR1; kindly donated by S. Ross), a hTfR1 expression vector (pcDNA3.1-hTfR1; kindly donated by P. Cannon) or pcDNA3.1 parental vector using the TurboFect transfection reagent (Thermo Scientific). Forty-eight hours post-transfection, the cells were either infected with wild-type MMTV(C3H) virus or transduced with *egfp*-containing MMTV-based vector particles carrying the WT or mutant (G42E) Env. Infection/transduction was assessed as described above.

## Additional files

Additional file 1: Figure S1. Infection of target cells with cell-free virus from supernatants of murine producer cells. (A) CHO cells transfected with hTfR1 or mTfR1expression plasmids were infected with cell-free virus from supernatants of murine producer cells [Mm5MT – MMTV(C3H)]. Expression of hTfR1 was confirmed by western blotting (insets); mTfR1 was not recognised by the α-TfR1 antibody (ab137944, Abcam). (B) Hs578T cells transfected with *hTFR* –specific siRNA or control siRNA were infected with cell-free virus from supernatants of murine producer cells [Mm5MT – MMTV(C3H)]. hTfR1 knock-down was confirmed by western blotting (inset). (A & B) Genomic DNA was isolated from infected cells one week post-infection and analysed by PCR for the presence of MMTV sequences. -ve: non-transduced Hs578T cells. +ve: producer cells. Equal DNA loading was controlled in the PCR assay with GAPDH-specific primers (bottom panels). Additional file 2: Figure S2. Truncated protein products resulting from the mutations introduced into the *hTfR1* locus using the CRISPR-Cas9 system.

## Abbreviations

MMTV: Mouse mammary tumour virus
TfR1: Transferring receptor 1
SU: Surface subunit
TM: Transmembrane domain
Sag: Superantigen
Rev: Regulator of virion expression
CA: Capsid
GP: Glycoprotein
Env: Envelope
pro: Protease
pol: Polymerase
gag: Group-specific antigen
HIV: Human immunodeficiency virus
MLV: Murine leukemia virus
JUNV: Junin virus
TCRV: Tacaribe virus
VSV-G: Vesicular stomatitis virus glycoprotein
CMV: Cytomegalovirus
FBS: Fetal bovine serum
GFP: Green fluorescence protein
DEX: Dexamethasone
RT-PCR: Real-time polymerase chain reaction
PCR: Polymerase chain reaction
PBS: Phosphate buffer saline
AZT: 3′-azido-3′-deoxythymidine
GAPDH: Glyceraldehyde 3-phosphate dehydrogenase
DNA: Deoxyribonucleic acid
RNA: Ribonucleic acid
siRNA: Small interfering RNA
LM-PCR: Linker mediated polymerase chain reaction
APOB: Apoliprotein B
GS: Glycosylation site
RBD: Receptor binding domain
RBS: Receptor binding site
HBD: Heparin binding domain
Pro-rich: Proline rich domain
NHEJ: Non-homologous end joining
HS: Herapan sulfate
FCM: Flow cytometry
WT: Wild-type
