# Supplementary material for: Single amino acid substitution (G42E) in the receptor binding domain of mouse mammary tumour virus envelope protein facilitates infection of non-murine cells in a transferrin receptor 1-independent manner
Source: Retrovirology. 2015 May 16;12:43. doi: 10.1186/s12977-015-0168-2 (PMC4445801; doi:10.1186/s12977-015-0168-2)
Supplement: Additional file 1: Figure S1. — Infection of target cells with cell-free virus from supernatants of murine producer cells. (A) CHO cells transfected with hTfR1 or mTfR1expression plasmids were infected with cell-free virus from supernatants of murine producer cells [Mm5MT – MMTV(C3H)]. Expression of hTfR1 was confirmed by western blotting (insets); mTfR1 was not recognised by the α-TfR1 antibody (ab137944, Abcam). (B) Hs578T cells transfected with hTFR –specific siRNA or control siRNA were infected with cell-free virus from supernatants of murine producer cells [Mm5MT – MMTV(C3H)]. hTfR1 knock-down was confirmed by western blotting (inset). (A & B) Genomic DNA was isolated from infected cells one week post-infection and analysed by PCR for the presence of MMTV sequences. -ve: non-transduced Hs578T cells. +ve: producer cells. Equal DNA loading was controlled in the PCR assay with GAPDH-specific primers (bottom panels). [file 12977_2015_168_MOESM1_ESM.ppt]

## Slide 1
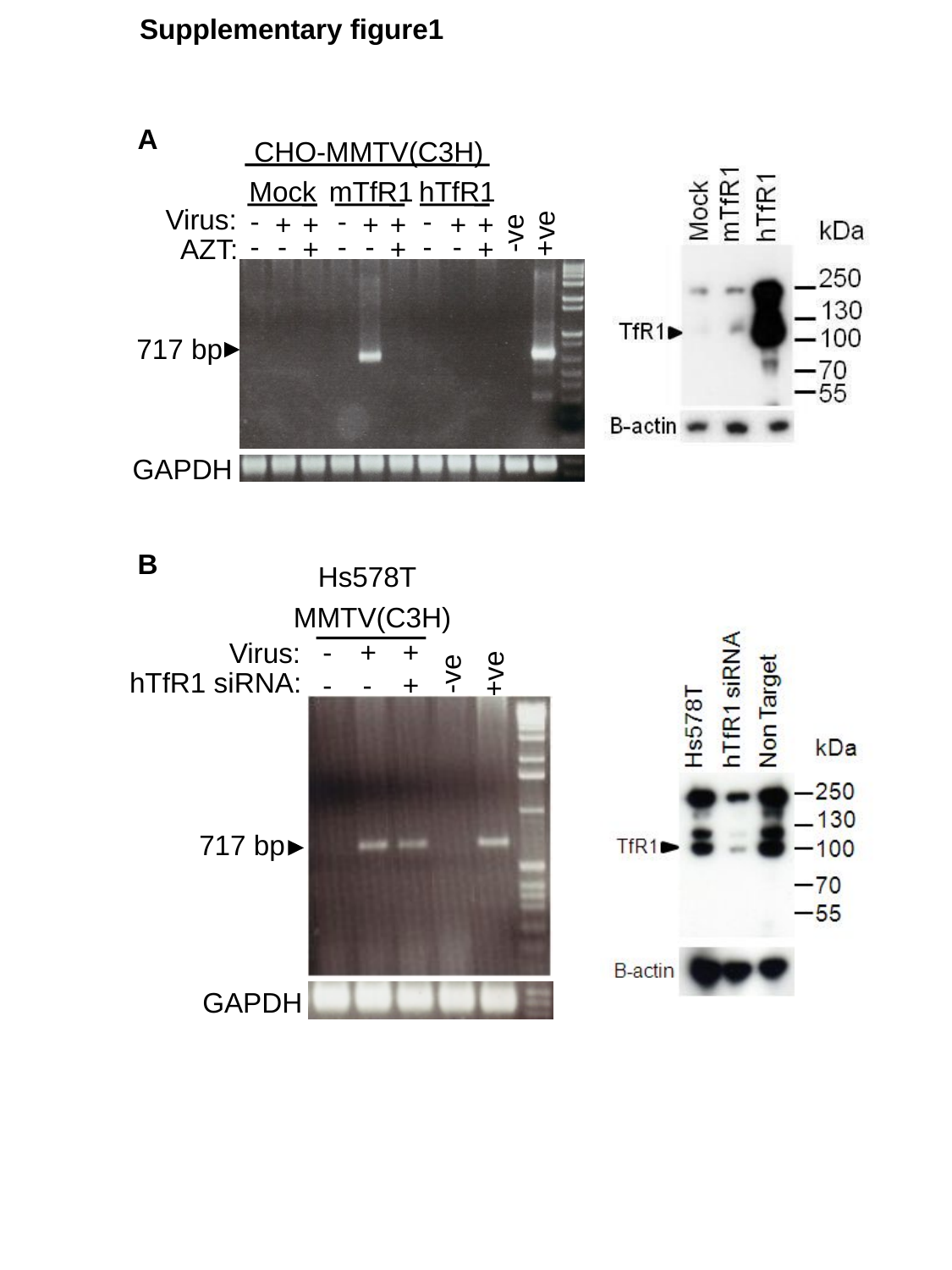

Supplementary figure1
A
CHO-MMTV(C3H)
Mock
mTfR1
hTfR1
Virus:
-
-
-
+
+
+
+
+
+
-ve
+ve
-
-
-
-
-
-
AZT:
+
+
+
717 bp
GAPDH
B
Hs578T
MMTV(C3H)
-
+
+
Virus:
-ve
+ve
hTfR1 siRNA:
-
-
+
717 bp
GAPDH
