# Supplementary figures and images for: Single amino acid substitution (G42E) in the receptor binding domain of mouse mammary tumour virus envelope protein facilitates infection of non-murine cells in a transferrin receptor 1-independent manner
Source: Retrovirology. 2015 May 16;12:43. doi: 10.1186/s12977-015-0168-2 (PMC4445801; doi:10.1186/s12977-015-0168-2)

## Slide 1
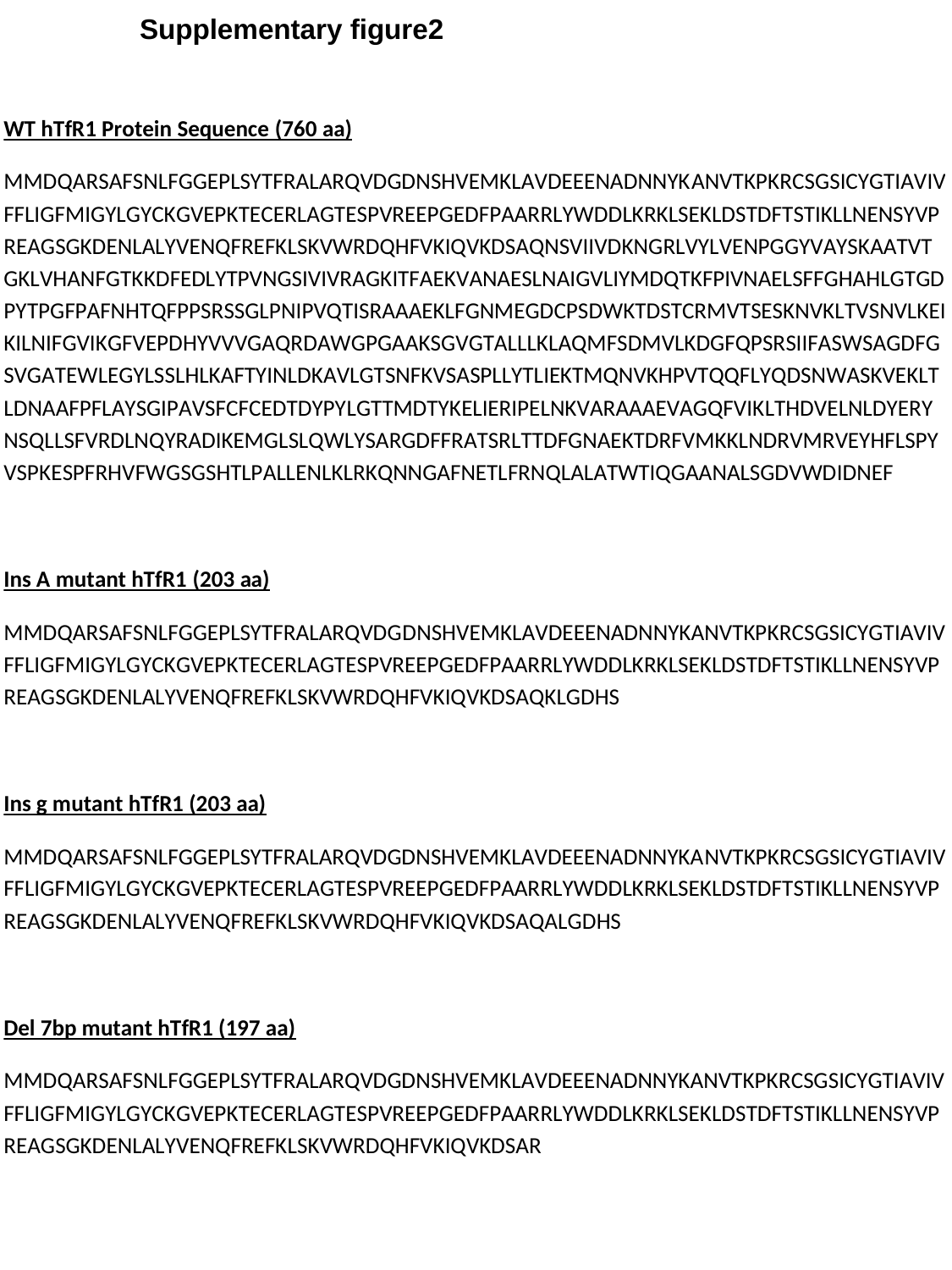

Supplementary figure2

Supplement: Additional file 2: Figure S2. — Truncated protein products resulting from the mutations introduced into the hTfR1 locus using the CRISPR-Cas9 system. [file 12977_2015_168_MOESM2_ESM.ppt]
